# An automatic immuno-microfluidic system integrating electrospun polystyrene microfibrous reactors for rapid detection of salivary cortisol

**DOI:** 10.1016/j.isci.2023.107820

**Published:** 2023-09-06

**Authors:** Yecan Wang, Hiroshi Murakami, Toshihiro Kasama, Shigenobu Mitsuzawa, Satoru Shinkawa, Ryo Miyake, Madoka Takai

**Affiliations:** 1 Graduate School of Bioengineering, The University of Tokyo, 7-3-1 Hongo, Bunkyo City, Tokyo 113-8654, Japan; 2 Honda Motor Co., Ltd, 8-1 Honcho, Wako, Saitama 351-0114, Japan

**Keywords:** Diagnostic technique in health technology, Analytical chemistry applications

## Abstract

Conventional competitive enzyme-linked immunosorbent assay (ELISA) to measure the cortisol level in body fluid consumes a large amount of time, owing to complicated operations involved and requirement for precise control of reagent addition. We developed an automatic microfluidic system to detect salivary cortisol rapidly, and an electrospun polystyrene (PS) microfiber-based reactor providing considerable binding sites for antibody immobilization, thus resolving the time limitations of competitive ELISA. Cortisol sample, horseradish peroxidase (HRP)-conjugated cortisol, and 3,3′,5,5′-tetramethylbenzidine (TMB) substrate were delivered to the PS reactor from containers in sequence by pumps automatically. The color variation due to oxidized TMB complex reflects the cortisol concentration level measured using an RGB phototransistor. In addition, the entire procedure from sample introduction to obtaining the photocurrent took only 15 min. This system can be implemented to quantify cortisol from 0.37 ng/mL to 30 ng/mL, and the limit of detection was estimated at 0.37 ng/mL.

## Highlights

• Rapid ELISA measurement using microfiber is four times faster than traditional assay
• Low-cost miniature RGB photodetector instead of expensive optical instruments
• Automatic operation enabling convenient process with reduction of technical mistakes
• Portable dimensions eliminating laboratory visit

## Graphical abstract

**Figure:**
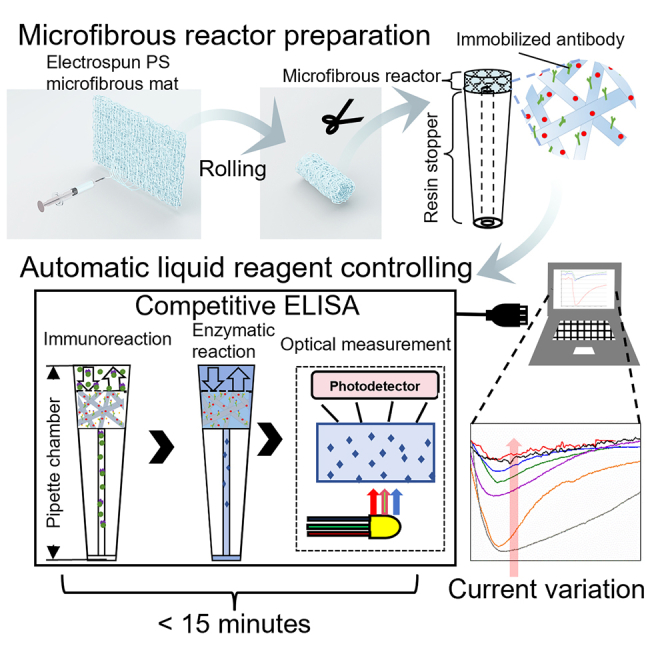

## Introduction

Cortisol, regarded as the stress biomarker, plays an essential role in reflecting health conditions. Cortisol secretion is controlled by the hypothalamic-pituitary-adrenal (HPA) axis, a principal contributor to the adaptive scheme of human body for preserving physiological processes.^1^^,^^2^^,^^3^ Cortisol regulates diverse physiological mechanisms, comprising carbohydrate metabolism, glucose concentration optimization, and maintaining blood pressure.^3^ Cortisol levels fluctuate throughout the day following a circadian rhythm that they summit in the morning and then gradually decrease until midnight.^4^ Abnormal cortisol secretion can depress the immune system, inhibit inflammation, and reduce fat and amino acid concentrations. Physiologically, excessive cortisol secretion can trigger Cushing’s syndrome which shows symptoms of fatigue, osteoporosis, and obesity. Deficiency of cortisol in the body may induce weight loss, exhaustion, abdominal pain, hypotension, and muscle weakness.^5^^,^^6^^,^^7^^,^^8^ Furthermore, cortisol is a classic biomarker indicating the stress state associated with physiological and psychological infirmity in the neuroendocrine system.^9^ Nowadays, people face stress from various aspects of society. When negative feedback caused by stress is received from routine behaviors, a rapid and reliable point-of-care (POC) immunoassay to evaluate cortisol levels is urgently required.

Due to the diffusion of cortisol through cells toward the blood circulation system, detectable amounts of cortisol can be identified in multiple biological samples. Cortisol in serum (30–230 ng/mL),^10^ sweat (8.16–141.7 ng/mL),^11^ hair (1.7 × 10^−3^ to 0.153 ng/mL),^12^ urine (10,000 to 100,000 ng/24 h),^13^ and saliva (2.2–27.3 ng/mL)^14^ have been reported in previous studies. In this study, saliva was selected as the target for POC devices owing to its intrinsic advantages compared with other body fluids. First, saliva mainly consists of various electrolytes, immunoglobulins, proteins, enzymes, mucins, and nitrogenous constituents (urea, ammonia, etc.).^15^ Second, there is a conspicuous relationship between cortisol levels in saliva and serum levels.^16^^,^^17^ Cortisol exists in a plasma-free state in the saliva.^13^ Finally, the non-invasive saliva collection technique has matured in the past decades, resulting in the simple collection of saliva by patients themselves at home.^15^

In recent years, typical immunosensing methods, including radioimmunoassay (RIA) and ELISA, to quantitatively analyze salivary cortisol have demonstrated high sensitivity and specificity,^18^ and ELISA is regarded as the gold standard technique for measuring analytes in aqueous target samples.^3^ However, the considerable time consumed by the process resulting from multiple steps, and the large scale of the detection apparatus are obstacles to diminishing the dimensions of the devices for POC application.^18^^,^^19^^,^^20^^,^^21^ Pinto and colleagues fabricated a polydimethylsiloxane (PDMS)-based microfluidic immunosensor to detect salivary cortisol levels using a competitive ELISA.^18^ The reagents were injected from three inlets into the microchannels created by lithography, followed by instant incubation in microwells immobilized with anti-cortisol antibody. The color-change solutions were conveyed to the integrated colorimetric analyzer by pumps. Although this immunosensor reduced the volume of reagents consumed, the measuring time, and the limit of detection (LOD), at least 35 min was required to complete the total procedure, as well as the restriction of complexity by the human hand. In the available ELISA kits, 96-well polystyrene microplates are usually used to immobilize antibodies. Generally, polystyrene (PS), as the ideal material for ELISA, is commercially used because of its non-toxicity, excellent biocompatibility,^22^ verified protein adsorption ability, which provides relatively steady hydrophobic bonds,^23^^,^^24^ low cost, and stable physicochemical properties.^25^ Furthermore, PS can be constructed into microfibers by electrospinning, which contributes to the micro-scale structure of the substrate with a high surface area and more binding sites for antibodies instead of the flat-structured well bottom. In our previous work, we reported that a high density of antibody binding on PS microfibers could achieve rapid immunoreaction.^24^^,^^26^ This study aimed to introduce a microfluidic system to operate automatically fluidic control changing the tedious manual procedures of conventional immunoassays to enable rapid measurement of salivary cortisol levels. This platform conducts the reaction in a PS microfibrous reactor with a three-dimensional (3D) structure, which replaces two-dimensional (2D) microplate wells. In addition to the automated delivery of liquid samples, this system enables rapid readouts in less than 15 min in contrast to the 35 min or more required by the competitive ELISA using well plate. Moreover, the simplicity of user operation and the briefcase size enables the system to be applied in self-testing at home and POC testing in clinics.

## Results and discussion

### Fabrication of anti-cortisol antibody immobilized microfibrous reactor

**Figure 1:**
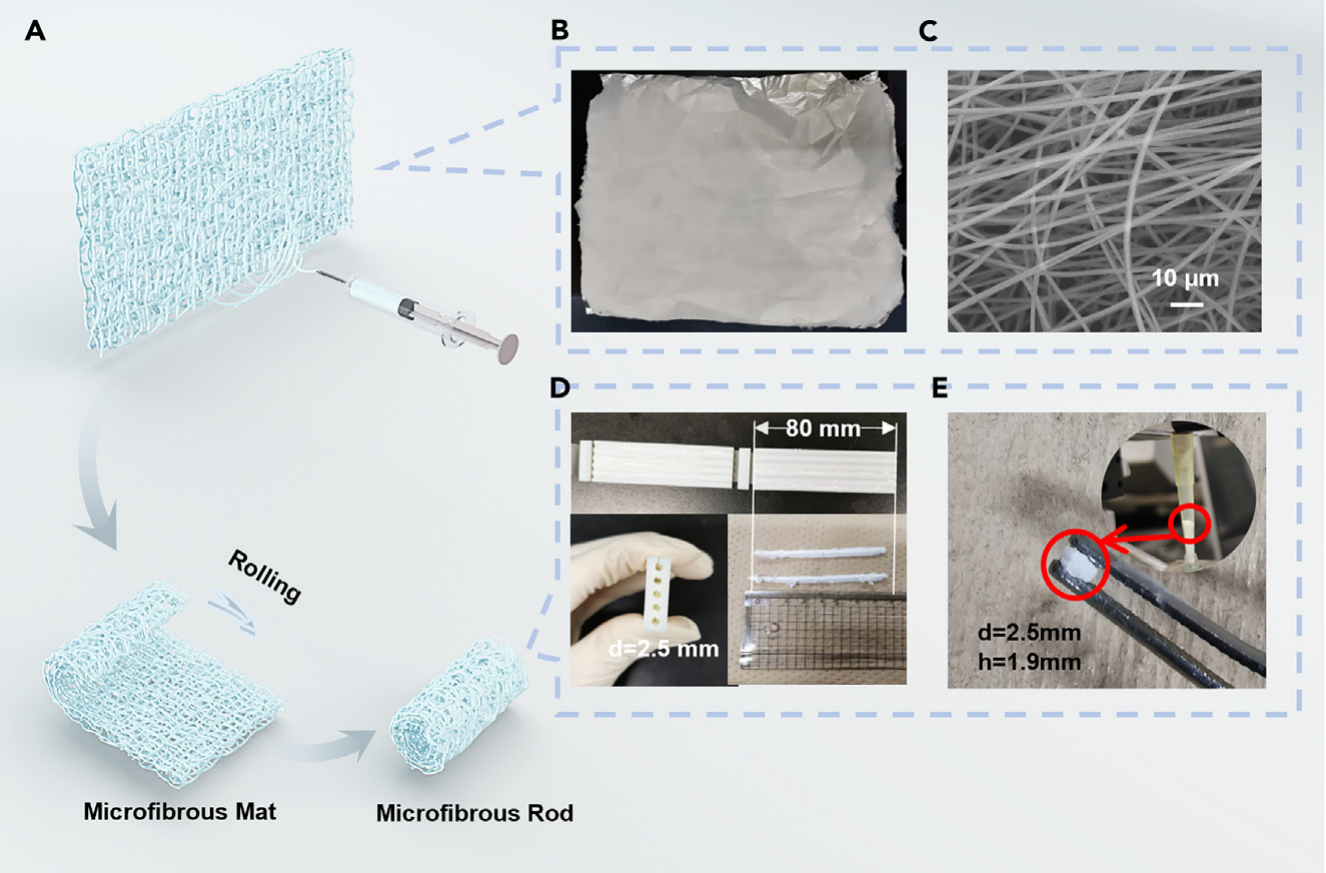
Schematic of the microfibrous reactor fabrication (A) Flow chart of microfibrous reactor fabrication from single layer microfibrous mat to microfibrous rod. (B) The photograph of electrospun single-layered microfibrous mat in the area of 30 cm × 40 cm on aluminum foil. (C) SEM image showing the morphology of the microfibrous mat surface. (D) The white resin-made mold (length 80 mm × diameter 2.5 mm) fabricated by the 3D printer, and the cylinder-like rod PS microfibrous mat reconstructed by the mold produced (on the bottom right). (E) The cropped white piece of “microfibrous reactor” which was later installed in a 200 μL pipette tip.

Figure 1A shows the procedure for rolling the microfibrous mats from the electrospinning technique drawn by the C4D software. We have fabricated the single-layered microfibrous mat in the area of 30 cm × 40 cm on aluminum foil shown in Figure 1B. The quality of the electrospun PS, which is mainly dependent on morphological homogeneity, was evaluated by SEM. These homogeneous microfibers without beads shown in Figure 1C ensured equivalent capacity for capturing antibodies and flow permeability. In Hoy and colleagues’ work, the number of proteins adsorbed on the surface of the microfibers was restricted by the surface area of the electrospinning mats.^26^ To increase the surface area, layers of microfibrous mats were stacked to prepare the immobilized antibody. According to their analysis, the 4-layered mat showed the best performance at 300 ng/well. In our study, we prepared the microfibrous rod by rolling up from a 70 μm single layer using a specific resin-made mold (length 80 mm × diameter 2.5 mm) fabricated by a 3D printer. The initial single layer thickness was estimated by the stacked microfibrous mats as described in Figure S1 and in method details. A cylinder-like matrix was constructed followed by curtailing to smaller-scale components, annotated as a “microfibrous reactor” (Figures 1D and 1E). These reestablished reactors contributed to a much greater volume than the layered mats, providing extraordinary binding sites for anti-cortisol antibody (Cat#GTX21949 RRID: AB_380320). Afterward, the microfibrous reactors were immersed in 10 μg/mL anti-cortisol antibody aqueous solutions for 2 h. Subsequently, the samples were rinsed in PBS for 1 h, followed by submersion in 1 wt % BSA blocking reagent for 15 min.

The capacity of the anti-cortisol antibody captured by the PS microfibrous reactor was evaluated according to the quantity of anti-cortisol antibody adsorbed. First, microfibrous reactors of various weights (0.5 mg, 0.6 mg, 0.7 mg, 0.8 mg, and 0.9 mg) were prepared to evaluate the anti-cortisol antibody capture capability of reactors possessing assorted pore sizes, and the fabrication method was described as follows. The microfibrous mat of various areas (8 cm × 7 cm, 8 cm × 8 cm, 8 cm × 9 cm, 8 cm × 10 cm, and 8 cm × 11 cm) were rolled up to prepare microfibrous reactors with assorted porous structures using the 3D printed mold (Figure 1D). These rolled microfibrous rods were then moisturized by ethanol in the mold, followed by placed overnight. Finally, these microfibrous rods fabricated by 8 cm × 7 cm, 8 cm × 8 cm, 8 cm × 9 cm, 8 cm × 10 cm, and 8 cm × 11 cm microfibrous mats were cropped into 2.5 mm × 1.9 mm reactors, and the weights were measured to 0.5 mg, 0.6 mg, 0.7 mg, 0.8 mg, and 0.9 mg, respectively. After the step-by-step procedure of antibody immobilization, the initial antibody solution, residual antibody solution, and washing buffer after rinsing were pipetted to measure the amount of protein using the micro-BCA assay. According to Equation 1 in method details, the antibody bound quantity in microfibrous reactors is shown in Figure S2A. The histograms indicated that 0.6 mg of the fabricated substrate showed the best performance for antibody capture, 0.37 μg Figures S2B–S2F show the SEM images comparing the different weights reactors for further study. When the weight increased, the density of the microfibrous reactor increased, resulting in the shrinkage of the pores. In addition, there were more aggregations in the sample of 0.9 mg than in the 0.6 mg from the SEM results. Another possible reason is that the fluids could not efficiently penetrate the shrunken pores in the microfibers. Furthermore, we used a confocal microscope (LSM 710, ZEISS) to observe the internal structure of the 0.6 mg microfibrous reactor. Figures S3A and S3B show the confocal microscopic reconstructed 3D projection images. The pore size was estimated using ImageJ (ImageJ, RRID:SCR_003070), and the distribution is summarized in the histograms in Figure S3C. According to these results, the pore sizes in the reactor were mainly concentrated in the range of 1–2 μm.

### Design and procedure of the microfluidic system

**Figure 2:**
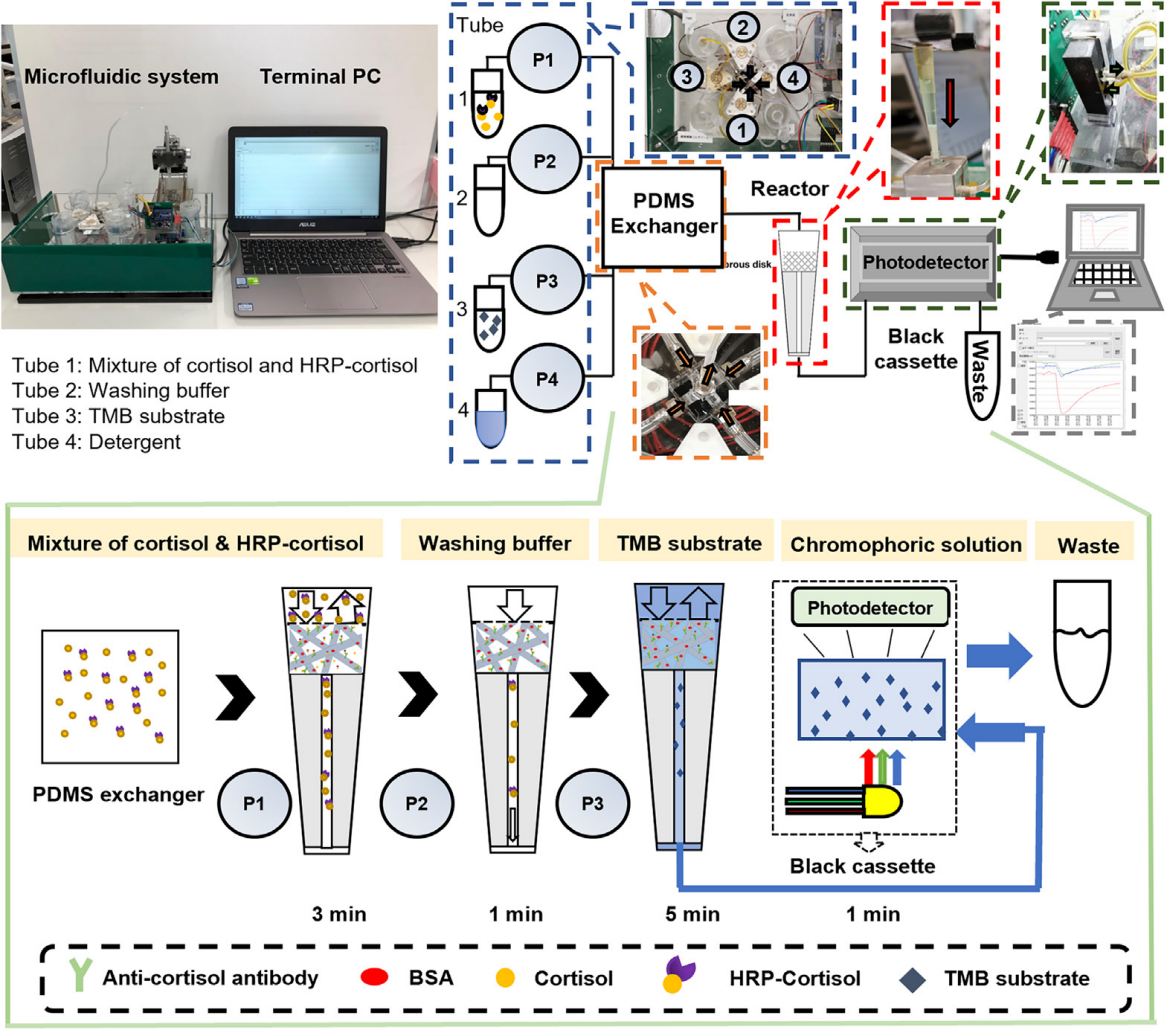
Photograph and schematic of the microfluidic system Set-up components are marked by dashed line: four reagent stock tubes, four pumps (blue dashed line), PDMS cubic exchanger (fluid exchanger made of PDMS) (orange dashed line), 200 μL pipette tip with a stopper installing the microfibrous reactor (red dashed line), black 3D-printed resin cassette enclosing RGB phototransistor (blackish green dashed line), signal output (gray dashed line). The schematic of the competitive ELISA via the automatic microfluidic system is also shown in the green box. In the competitive procedure of HRP-cortisol/cortisol incubation, and TMB incubation, the flow direction in the pipette chamber is switched for mixing it up and down (255 μL/min). A colored arrow marked the flow direction. See also Figures S4–S7.

Figure 2 shows the photograph and schematic of the microfluidic system, the components of which can be observed in Figure S4. Four 1.5 mL centrifuge tubes (Tube 1–4) were used to store the reagents. Additionally, four pumps (Takasago Electric, Inc.), denoted as P1–P4, were used for liquid operation. The reagents were connected using 0.7 mm diameter of Tygon-made tubes. These pumps generate power to convey reagents into the chamber for fluid exchange made of PDMS. The reagents were then driven toward a 200 μL polypropylene (PP) pipette tip (epT.I.P.S. Standard, Eppendorf Corp., Germany), where the microfibrous reactor was installed through a Tygon tube. As shown in Figure S5, a 3D-printed stopper with a size fitted to the pipette tip was used to stabilize the reactor. A channel with a diameter of 1 mm was penetrated to allow microfluidics to concentrate through the stopper.

The measurement method was described as follows. After starting the system, the addition of HRP-cortisol and cortisol solutions at a determined ratio were transferred into the microfibrous reactor through the PDMS exchanger to participate in the competitive ELISA reaction in the microfibrous reactor for 3 min (P1). The washing buffer was then delivered to wash out excess reagents for 1 min (P2), followed by incubation in the TMB substrate for 5 min (P3). The operation control circuit board was assembled using an Arduino board and then connected to our terminal device. The fluid was operated using the developed software in the C++ language. The feeding rates of the reagents could be set from 0 to 255 μL/min. Furthermore, four feeding functions named: “Push,” “Back,” “Alternate,” and “Waiting” were available for pushing fluids forward, returning fluids backward, fluids running back and forth alternately, and suspending, respectively (Figure S6). In this study, the “Alternate” mode was adopted in the incubation procedures, the antigen-antibody binding competition (anti-cortisol antibody and HRP-conjugated cortisol and/or cortisol), and enzyme-substrate reaction (HRP-conjugated cortisol and TMB) to accelerate the efficiency. The colorimetric level attributed to the enzyme-substrate reaction was detected using an RGB photodetector (NJL7502L) purchased from Akizuki Denshi Tsusho Co. Ltd. The photodetector was enclosed in a black cassette to prevent external light exposure (Figure S3). Briefly, the photodetector as shown in Figure 2 consists of a white LED and a photoresistor. Red, green, and blue light were alternately emitted through the solution from the LED source. A portion of the light, specifically red light, was absorbed by the solution during penetration, depending on the color of the solution and the intensity of the color. The transmitted light illuminated on the photoresistor causing a change in the current signal and was then output to the terminal computer. After the measurement, some reagents are residual on the surface of the interior walls of the system (e.g., conducting tubes and the PDMS exchanger), thus the P4 was applied to transmit the detergent to rinse the whole system to accomplish the self-cleaning function.

In addition, we measured the heights of three microfibrous reactors before and after experiment. The original heights were 1.91 mm, 1.86 mm, and 1.86 mm, respectively. After the reaction, the heights were measured at 1.90 mm, 1.86 mm, and 1.84 mm, respectively. Thus, the ratios of variation were 0.5%, 0, and 1.1%, indicating that the microfibrous reactors could maintain the microporous structure during the test in the microfluidic system.

### Data processing

**Figure 3:**
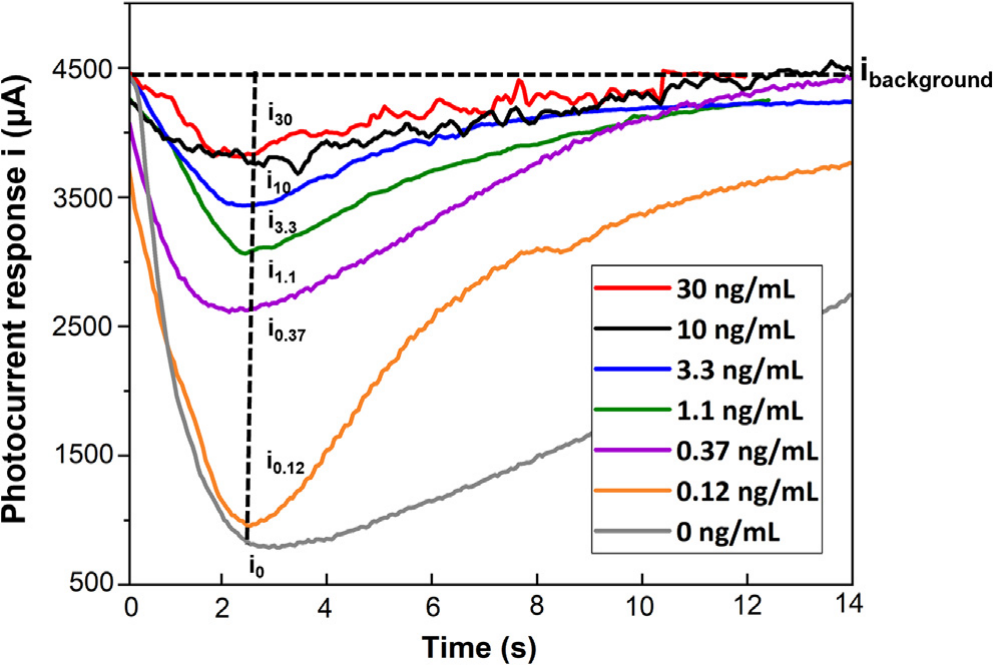
Signal output of this photodetector The current response reflecting color change degrees in standard solutions with various cortisol concentrations (0 ng/mL, 0.12 ng/mL, 0.37 ng/mL, 1.1 ng/mL, 3.3 ng/mL, 10 ng/mL, and 30 ng/mL). These signal profiles plotted according to n = 1 independent measurement for each concentration.

The analysis of cortisol detection using this microfluidic system is fundamental to color intensity. Theoretically, the TMB substrate, purchased from Abcam Co., was oxidized and generated a blue charge-transfer complex due to the enzymatic degradation of hydrogen peroxide by HRP. In competitive ELISA, HRP-cortisol from the ELISA kit (YK241 Cortisol EIA Kit, Yanaihara Institute Inc.) and cortisol molecules were in a competitive relationship when binding to the antibody, indicating the color intensity was inversely to the cortisol concentration. Figure 3 shows the representative response for various cortisol concentrations using 150 μL HRP-cortisol solution and 50 μL cortisol solution with different concentrations. HRP-cortisol solution and the cortisol solutions were used from the commercial competitive ELISA kit (YK241). The cortisol solutions were prepared as 0.12, 0.37, 1.1, 3.3, 10, and 30 ng/mL from standard solutions.150 μL of HRP-cortisol solution and 50 μL of cortisol solution were prepared and then mixed in tube 1. The 200 μL mixture solution was delivered to participate in the competitive ELISA automatically, and various current responses corresponding to the concentrations were generated.

We can see that the signal profile for lower cortisol concentration required a longer time for recovery after passing the peak value, resulting from more residuals of blue TMB complexes produced during the reaction between the TMB substrate and conjugated HRP. Applying Equation 1, the cortisol concentration is determined by referring to the variation ratio of photocurrent (*I*) according to the calibration curve, which represents the color indicator inversely proportional to the concentration of cortisol:

(Equation 1) $$I=\frac{\Delta i}{i_{backgraound}}=\frac{i_{background}-i}{i_{background}}$$

where *i* and *i*_*background*_ are the current responses of the target and background substrate solution, respectively. In this study, TMB substrate was selected as the background solution for the colorimetric reaction.

In competitive ELISA, the index *B/B*_*0*_ is a parameter of assay system performance. *B* is the binding signal between the antibody and antigen, and *B*_*0*_ is the signal of the sample without antigen. Here, *B/B*_*0*_ can be estimated using Equation 2:

(Equation 2) $$\frac{B}{B_{0}}=\frac{I}{I_{0}}=\frac{i-i_{background}}{i_{0}-i_{background}}$$

where *i*_*0*_ is the current signal from the sample solution containing no cortisol analytes.

### Mass transport in the microfibrous reactor

As described in previous contents, a unique flowing mode called “Alternate mode” has been used in the incubation process of HRP-cortisol and cortisol competition for binding with anti-cortisol antibody and the process of TMB substrate and HRP enzyme reaction. This mode enabled microfluidics to transverse back and forth in the microfibrous reactor to accelerate concentration diffusion and expedite conjugation reactions. The velocity of the solute intra- and inter-fiber fractures, along and across the flow direction, is determined by the diffusion and advection velocities.^27^ In other words, the diffusion and advection velocities of mass transport co-impact on solute transfer velocity. Owing to the complex porous structure inside the microfiber, the flow path of the fluid was irregular, and the fracture size was not homogeneous. When the fluid flows through the microfibrous reactor, a reversal flow is used to bring the solute more fully in contact with the microfiber walls. Another reason is that the reversal flow can also increase the diffusion rate of solutes inside the microfiber fractures. Taylor-Aris dispersion is regarded as the classic analytical method for studying and evaluating the dispersion of solutes in laminar flows.^28^^,^^29^^,^^30^ The viewpoint of this approach is that the mean concentration of the solute forms a symmetric Gaussian distribution in the longitudinal direction after an adequate amount of time, during which the effective diffusion coefficient is greater than the molecular diffusion coefficient. However, the axial concentration diffusion in a unidirectional fluidic system shows significant asymmetry because the diffusion is aligned with the advection downstream. In contrast, the reversal advection upstream hinders the diffusion.^31^ Furthermore, Wang and Chen investigated the diffusion effect of pollutants in a reversal flow, where the concentration contours were uniformly distributed both upstream and downstream during a reversal period.^32^ When switching the fluid orientation from downstream to upstream, the concentration contours reverse as the velocity profile results in a significant concentration difference in the areas covered by the contours and vice versa. Thus, the dispersion of the solute was reinforced, leading to a shorter time for the molecules to reach the solid surface immobilized with the antibody. Figure S7 compared the effect of different HRP and TMB incubation times on the output signal in the static mode (flow rate was zero) and in the “Alternate mode” (reversal flow with the rate of 255 μL/min). It could be observed that the “Alternate mode” effectively increased the reaction efficiency between the HRP enzyme conjugated on the surface of the microfibers and the TMB substrate in the fluid. Also, we could conclude that 5 min was the optimal operation time in the TMB-HRP reaction. In conventional ELISA kit, this step might take 30 min due to the flat microplate wells with much lower surface area compared to 3D structured microfibrous reactors. Consequently, the entire procedure, from sample introduction to obtaining the photocurrent of the reaction, took only 15 min.

### Comparison between conventional competitive ELISA using a 96-well plate and the developed microfluidic system

Three ratios of HRP-cortisol and the sample containing cortisol were prepared to determine the optimal ratio of HRP-cortisol solution and standard solution with various cortisol concentrations. Three replicates were analyzed for each test. For comparison, the standard curves of commercial competitive ELISA kits were also plotted and fitted using 5-parameter logistical regression (5PL) expressed as (Equation 3):

(Equation 3) $$y=d+\frac{a-d}{{\left[1+{\left(\frac{x}{c}\right)}^{b}\right]}^{g}}$$

where *a* and *d* are the theoretical values of B/B_0_ at zero and infinite cortisol concentrations, respectively. *b* represents the hill slope factor, *c* is the mid-range concentration, and *g* is the asymmetry factor.

**Figure 4:**
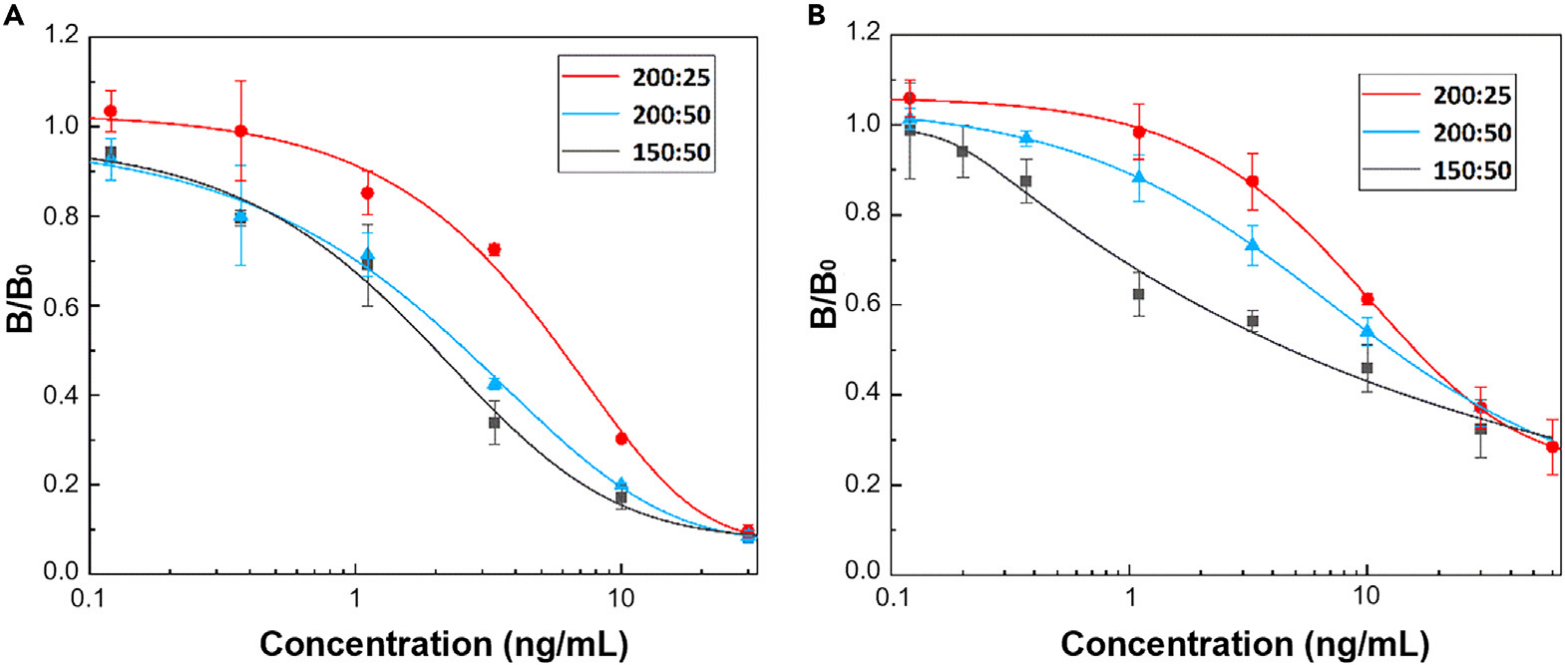
Plotted standard curves fitted according to various cortisol concentrations (A) Standard curves obtained by commercial competitive ELISA kit with HRP-cortisol/cortisol solution of 150 μL/50 μL (150:50), 200 μL/25 μL (200:25), and 200 μL/50 μL (200:50), respectively. (B) Standard curves obtained by the microfluidic system immunoassay with HRP-cortisol/cortisol solution of 150:50, 200:25, and 200:50, respectively. Error bars: standard error of the mean from n = 3 independent measurements.

In this study, the cortisol concentrations were used as 0.12, 0.37, 1.1, 3.3, 10, and 30 ng/mL for making the standard curves of the conventional ELISA kit using 96 well plate and our microfluidic system. The 50 μL or 25 μL volumes of cortisol solutions with different concentrations were mixed with the volume of 150 μL, or 200 μL of HRP-cortisol solutions for the evaluation of competitive reaction on microfibrous reactor. The standard curves were shown in Figures 4A and 4B. Comparing the standard curves of competitive ELISA (Figure 4A), the variations in the standard curves with three HRP-cortisol fractions using the microfluidic system (Figure 4B) were consistent: the linear ranges were observed to be right-shifted and more confined with the rising HRP-cortisol fraction. Table S1 shows the *B/B*_*0*_ and standard deviations obtained through the microfluidic system for standard curves plotting using three conditions for HRP-cortisol to cortisol ratios, 150 μL:50 μL, 200 μL:50 μL, and 200 μL:25 μL. In these three conditions, the relative standard deviations (RSD) were almost calculated within 10%, indicating the measuring results were reliable. As shown in Table S2, the fitted curves obtained from the commercial ELISA kit in Figure 4A converged at approximately 0.07 (*d* = 0.07), whereas the *d* values obtained using the microfluidic system in Figure 4B (Table S2, *d* = 0.05–0.23) were positively correlated with the HRP-cortisol concentration. This result might be attributed to the microfibrous structure, in which some HRP enzymes were caught physically. Among the three reagent volume conditions in Figure 4B, the calibration curve with 200 μL of HRP-cortisol solution and 50 μL of the sample displayed the best fitting because of the correlation coefficient (*R*^*2*^) of close to 1, as shown in Table S2. Hence, a 200 μL:50 μL ratio was selected to measure the actual salivary cortisol levels in the following experiments.

The limit of detection (LOD) is an essential parameter for evaluating the performance of an immunoassay. The LOD represents the smallest concentration detectable by the system. Following the criteria of the International Union of Pure and Applied Chemistry (IUPAC),^33^ the LOD could be determined using Equation 4 based on blank samples.

(Equation 4) $$LOD=\frac{3.3\times SD}{Slop\;oflinearity}$$

Where *SD* is the standard deviation of the blank samples. With the selected HRP-cortisol/cortisol samples of 200 μL/50 μL, the linear regression equation was *B/B*_*0*_ = −0.3207log(*C*) + 0.8668 (*R*^*2*^ = 0.9805), where the slope of linearity was 0.3207. Therefore, this microfluidic system’s LOD was estimated at 0.37 ng/mL, close to that of the commercial ELISA kit, estimated at 0.16 ng/mL. The linear range in this condition was 0.37–30 ng/mL.

### Selectivity of the microfibrous reactor

**Figure 5:**
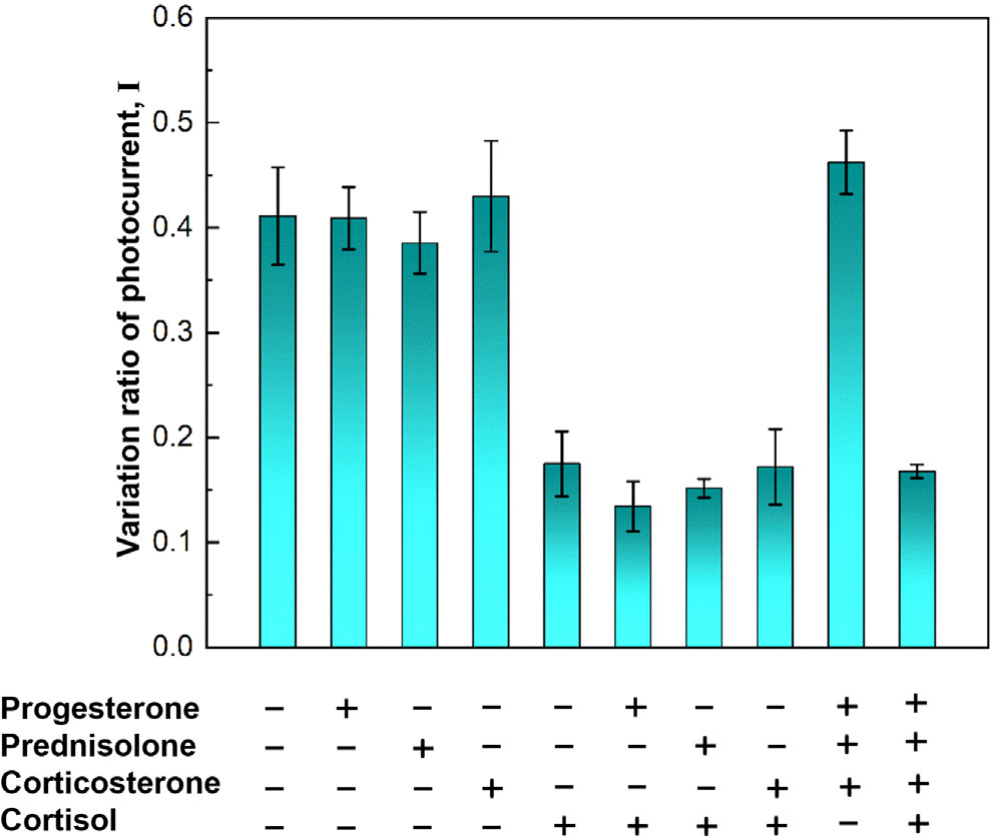
Selectivity of microfluidic system with antibody immobilized microfibrous reactors Three hormones with similar structure to cortisol were prepared to evaluate interference of various hormones, where the artificial saliva was used as the control group. Plus (+) and minus (−) signs on the X axis represent the presence and absence of some element, respectively. Error bars: standard error of the mean from n = 3 independent measurements.

The specificity test of the microfibrous reactors was performed using progesterone, prednisolone, and corticosterone, which have a structure similar to that of cortisol. These interfering hormones served as the control group at 100 ng/mL concentration. Solutions of these steroid hormones dissolved in artificial saliva^34^ (50 μL) were individually mixed with 200 μL of HRP-cortisol and then operated using this microfluidic system. Figure 5 shows the histograms of the selectivity for interfering hormones. Except for solutions containing cortisol, the intensity of these hormones exhibited an insignificant difference at a concentration of 100 ng/mL. It can be concluded that the anti-cortisol antibody immobilized microfibrous reactors are specific to cortisol.

### Detection in actual saliva

To evaluate the functionality of the microfluidic system with microfibrous reactor using actual saliva, both the conventional ELISA kit and the fabricated platform were tested using the mixing solution of 50 μL saliva sample and 200 μL BSA diluted HRP-cortisol solution. The salivary cortisol in saliva samples were harvested by two volunteers every morning (8 a.m.–10 a.m.) and evening (10 p.m.–12 a.m.) on three successive days. The volunteers were required to rinse their mouths with water 30 min before each harvest. The SalivaBio Oral Swab (Salimetrics, LLC., CA, USA), a synthetic swab specifically designed to improve volume collection, was then placed under the tongue, and held for 1 min 30 s, followed by preservation in a swab storage tube (Salimetrics, LLC., CA, USA) at −20°C. Alcohol consumption was prohibited for the duration of the saliva collection. The tools used to collect saliva samples are shown in Figure S8.

**Figure 6:**
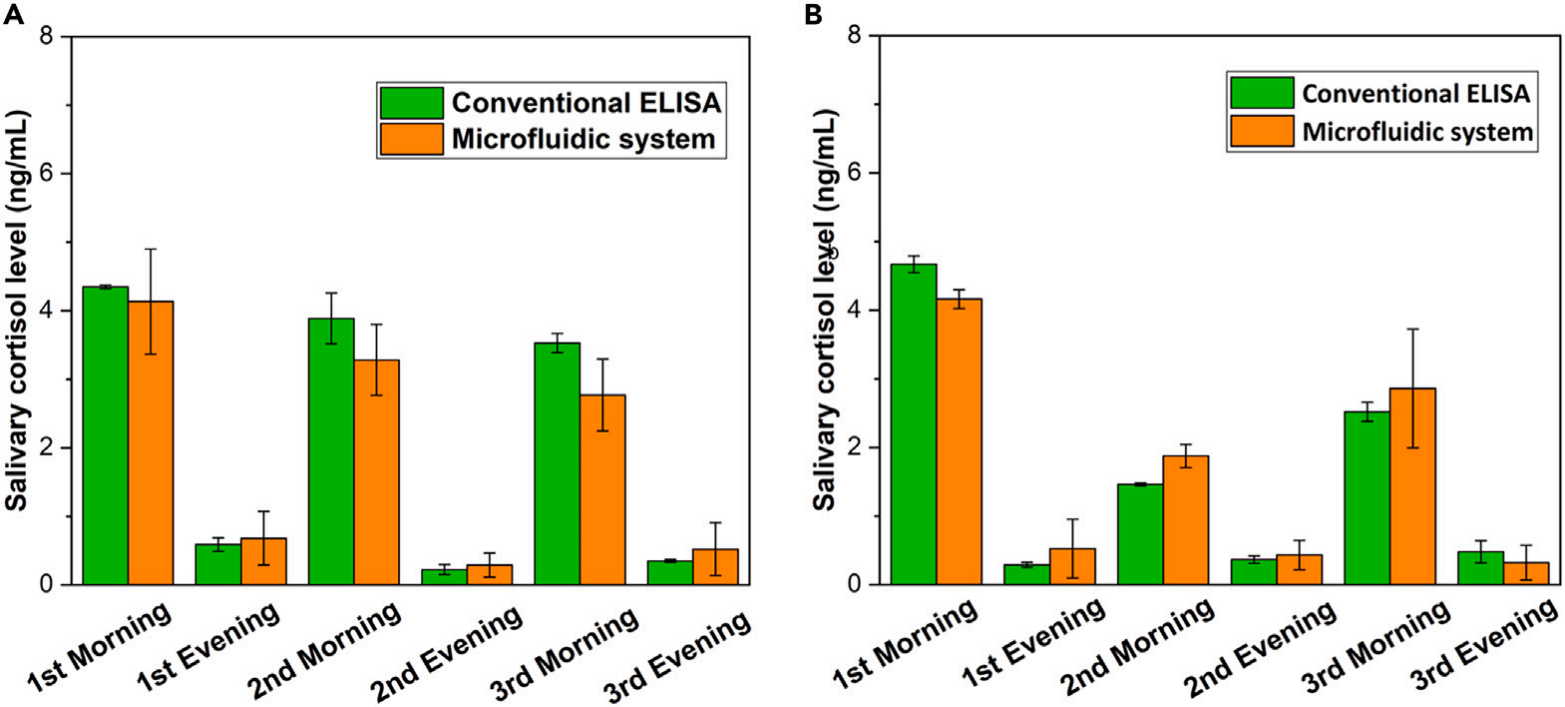
Performance of actual saliva sample measurement (A and B). Cortisol levels in actual saliva samples collected from two volunteers in the morning and evening measured by a competitive ELISA kit and the microfluidic system. Error bars: standard error of the mean from n = 3 independent measurements.

The standard curves are shown in Figures 4A and 4B, where the profiles of 150:50 and 200:50 were applied in the actual salivary cortisol measurement experiment by conventional competitive ELISA and the microfluidic system, respectively. From Figures 6A and 6B, salivary cortisol levels were higher in the morning than in the evening, corresponding to the diurnal circadian rhythm reported in previous studies.^4^^,^^13^^,^^16^^,^^35^ The results obtained from this microfluidic system were similar to those from the ELISA kit, implying that this system enables detection in actual saliva samples.

### Conclusion

An automatic immuno-microfluidic system was successfully assembled to detect saliva cortisol levels rapidly. This apparatus employs a flowing mechanism that can simplify the tedious procedures in typical immunoassays and optimize the detection time to 15 min. In this study, a unique disk-like reactor fabricated by an electrospinning technique was found to be superior to the typical microplate owing to its large surface area, excellent biocompatibility, robust structure, and high antibody capture capacity. In addition, the operating range, 0.37–30 ng/mL, was obtained from 5PL regression for the standard curve in accordance with the commercial competitive ELISA kit. We also concluded that this rapid detecting assay demonstrated a similar LOD of 0.37 ng/mL compared with 0.16 ng/mL of the ELISA kit. In addition, this immunoassay exhibits remarkable selectivity for cortisol among hormones with similar structures. Finally, we applied this microfluidic system to detect cortisol levels in actual saliva samples and a result comparable to that of the ELISA kit was obtained. Therefore, our study has successfully developed a microfluidic system capable of directly measuring cortisol levels in saliva, as well as the potential for *in vitro* detection of other targets such as hormones, metabolites, and even virus. In the future, we would like to implement this apparatus in health monitoring and POC diagnosis in the clinic for a broad range of applications.

### Limitations of the study

By integrating microfiber-based reactor and controllable microfluidic system, the sensitivity of the automatic immuno-microfluidic system is comparable to conventional competitive ELISA. However, its sensitivity can nevertheless be improved through a more homogeneous fibrous structure and an optimum blocking treatment. Also, highly viscous body fluids such as saliva are required to enhance flowability and prevent uneven mixing in microfluidics. In addition, the whole scale of the system is currently designed to a briefcase size, and we would like to further miniaturize it for more portable.

## STAR★Methods

### Key resources table

**Table:** 

REAGENT or RESOURCE	SOURCE	IDENTIFIER
**Antibodies**		

Anti-Cortisol, Mouse-Mono [XM210]	GeneTex	Cat#GTX21949; RRID: AB_380320

**Biological samples**		

Human’s saliva	Students from the University of Tokyo	N/A

**Chemicals, peptides, and recombinant proteins**		

Tween 20	Sigma-Aldrich	P1379
Bovine serum albumin	Sigma-Aldrich	1002586561
Polystyrene pellets	PS Japan Co. Ltd.	SGP-10
Tetrahydrofuran (THF)	FUJIFILM Corp.	CAS RN®: 109-99-9
N,N′-dimethylformamide (DMF)	FUJIFILM Corp.	CAS RN®: 68-12-2
Phosphate-buffered saline (10×)	FUJIFILM Corp.	163–25265
Corticosterone	FUJIFILM Corp.	CAS RN®: 50-22-6
Prednisolone	FUJIFILM Corp.	CAS RN®: 50-24-8
Progesterone	FUJIFILM Corp.	CAS RN®: 57-83-0
3,3′,5,5′-tetramethylbenzidine (TMB) substrate	Abcam Co.	ab171522
Mucin, from porcine stomach	FUJIFILM Corp.	CAS RN®: 84082-64-4

**Critical commercial assays**		

Cortisol ELISA kit	Yanaihara Institute Inc.	YK241
MicroBCA™ protein assay kit	Thermo Scientific	WK335032

**Software and algorithms**		

Cinma 4D R23	MAXON	https://www.maxon.net/ja/downloads/cinema-4d-r23-downloads
Origin 2018	OriginLab	https://www.originlab.com/2018 RRID: SCR_014212
ImageJ	NIH	https://imagej.nih.gov/ij/download.html RRID: SCR_003070

### Resource availability

#### Lead contact

Further information and any related requests should be directed to and will be fulfilled by the lead contact, Madoka Takai (takai@bis.t.u-tokyo.ac.jp).

#### Materials availability

All materials are in the key resources table. This study did not generate new unique and reagents. There are restrictions to the availability of the human saliva samples due to ethical privacy guidelines in this study.

### Experimental model and study participant details

No biological samples were from patients or subjects other than the involved authors in this study, and saliva samples were obtained from the two male co-authors. There were no ethical concerns involved in this study. The biological samples were collected in three days consecutively (6 samples/person). They were required to rinse their mouths with water 30 min before each harvest. The SalivaBio Oral Swab (Salimetrics, LLC., CA, USA), a synthetic swab specifically designed to improve volume collection, was then placed under the tongue, and held for 1 min 30 s, followed by preservation in a swab storage tube (Salimetrics, LLC., CA, USA) at −20°C. Each tube collected around 2 mL saliva samples. The tools are shown in Figure S8. Alcohol consumption was prohibited for the duration of the saliva collection. These saliva samples were used only for ELISA and practicability of our microfluidic system, where biological information is kept confidential. Therefore, the study also did not involve any research on sex and gender, ancestry, race or ethnicity.

### Method details

#### Fabrication of PS microfibrous reactors

The Polystyrene (PS) microfibrous mats were fabricated according to a previous study.^26^ PS pellets (10% v/v) were dissolved in N,N-dimethylformamide (DMF)/tetrahydrofuran (THF) (1:1, v/v) containing 2 wt. % Tween 20 as a surfactant. The PS solution was loaded into a 20 mL syringe (Terumo, Co., Tokyo, Japan), enclosed by a 21-gauge needle (Terumo, Co., Tokyo, Japan) as the spinneret, to fabricate microfibrous mats via electrospinning (NEU, Kato Tech Co., Ltd). The solution was fed at a flow rate of 0.167 mL/min, and a voltage of 25 kV was applied to the drum collector (d = 100 mm, L = 300 mm) covered with aluminum foil. The needle tip was placed approximately 18 cm away from the collector. The entire electrospinning procedure was performed at a relative humidity of 60%. Microfibrous mats with a single layer of approximately 70 μm fabricated on the tinfoil were then placed in a vacuum drier to eliminate residual solvents. Afterward, 8 cm × 8 cm microfibrous mats were reeled in a specific resin-based mold (length 80 mm × diameter 2.5 mm), which was prepared via a 3D printer (Eden260VS, Stratasys, MN, USA) to construct the column-like shape by the ‘wet press’ technique. The reeled microfibrous mats aligned in the mold grooves were immersed in ethanol and then pressed for 24 h. The column-like microfibers were cut into tiny components with a length of 1.9 mm in a microfibrous reactor. These components were annotated as microfibrous reactors waiting for antibody pre-treatment before use.

#### Measurement of single-layer microfibrous mats thickness

We prepared multiple stacked layers to measure the thickness and then calculated the thickness of one single layer. First, we took one microfibrous mat and folded it for five times to get 32-layered one. We then compacted the two microfibrous mats (64 layers in total) using the ‘wet press’ method. The ‘wet press’ is the method to increase the thickness of microfibrous mat. The procedure is to stack the microfibrous mats by multiple layers and then moistened with alcohol. The stacked wet mats are then sandwiched in two pieces of glass slice, on which the weights were put to compact of the microfibers. After compressing, the folded microfibrous mats were dried, and the thickness was then measured with a vernier caliper (A&D Company Limit, resolution = 0.01 mm). The average thickness per layer was obtained through dividing by the number of layers 64.

Figures S1A–S1D showed the SEM images of microfibrous mats compacted by various weights (0.1 kg–3 kg). We can see that the structures of microfibrous mats pressed by 1 kg and 3 kg weights were denser compared with that pressed by 0.1 kg and 0.5 kg. Evidently, the pores of the microfibers are most compacted by 3 kg weight. Figure S1E was the estimated thickness of single layer microfibrous mats thickness. The initial thickness of single layer was estimated around 70 μm.

#### Immobilization of anti-cortisol antibody in PS microfibrous reactors

The prepared microfibrous reactors were immersed in ethanol for 5 min, followed by immersion in DI water for 1 min. Then, a 10 μg/mL anti-cortisol antibody (GeneTex, CA, USA, Cat#GTX21949) solution was prepared by diluting 7 mg/mL dope using 1×PBS buffer (pH = 7.4), where the wet microfibrous reactors were submerged at room temperature for 2 h. After immobilization, the solid media were rinsed in 1×PBS for 1 h to remove excess unbound antibodies. These microfibrous reactors with antibody immobilization were applied in a single use.

#### Quantification of immobilized anti-cortisol antibody on microfibrous reactor via Micro-BCA assay

We studied the anti-cortisol antibody adsorbed on PS microfibers using a Micro bicinchoninic acid assay kit (Micro-BCA, Thermo Scientific, USA), which can quantitatively determine the amount of protein by a colorimetric assay. Antibody-immobilized microfibrous reactors with various weights were prepared individually by immersion in 10 μg/mL anti-cortisol antibody solution for 2 h, followed by soaking in PBS to rinse unbound antibodies for 1 h. Supernatants before and after antibody immobilization, and the PBS washing buffer after rinsing were pipetted into 96-well microplate wells. The micro-BCA method was then applied to these pipetted aqueous samples to quantify the anti-cortisol antibody in each sample. The calculation formula for the quantity of antibody immobilization is as (Equation 5):

(Equation 5) $$M={[C}_{i}-\left(C_{r}+C_{w}\right)]\times V_{l}$$

where *M* is the amount of antibody immobilized (μg), *C*_*i*_ is the anti-cortisol antibody concentration of the initial solution before immobilization, *C*_*r*_ is the residual anti-cortisol antibody concentration after immobilization, *C*_*w*_ is the anti-cortisol antibody concentration of the PBS washing buffer after washing, and *V*_*l*_ is the volume of the sampling liquid.

#### Conventional competitive ELISA experiment

The conventional ELISA experiment used a competitive ELISA kit (YK241 Cortisol EIA Kit, Yanaihara Institute Inc., Japan) in 96 well plates. Standard solutions of six different concentrations (0.12 ng/mL, 0.37 ng/mL, 1.1 ng/mL, 3.3 ng/mL, 10, and 30 ng/mL) were prepared to generate the standard curve.

#### Preparation of artificial saliva

Artificial saliva was prepared by dissolving 0.33 g/L KH_2_PO_4_, 0.34 g/L Na_2_HPO_4_, 1.27 g/L KCl, 0.16 g/L NaSCN, 0.58 g/L NaCl, 0.17 g/L CaCl_2_, 0.16 g/L NH_4_Cl, 0.2 g/L urea, 0.03 g/L glucose, 0.002 g/L ascorbic acid and 2.7 g/L porcine mucin in deionized water and then adjusted pH to 7.0.^34^ The deionized water used in the above experiment was processed using a water purification system (Milli-Q).

### Quantification and statistical analysis

We used Origin 2018(9.5) for data visualization to generate the standard curves using five-parameter logistic regression. We used absolute values or mean +/− standard deviation to express the results. The sample number n was stated in the relevant figure legends.

## Data Availability

• Data reported in this paper will be shared by the lead contact upon request. • This study does not use any code. • Any additional information required to reanalyze the data reported in this paper is available from the lead contact upon request.
