# An automatic immuno-microfluidic system integrating electrospun polystyrene microfibrous reactors for rapid detection of salivary cortisol

**DOI:** 10.1016/j.isci.2025.113252

**Published:** 2025-08-06

**Authors:** Yecan Wang, Hiroshi Murakami, Toshihiro Kasama, Shigenobu Mitsuzawa, Satoru Shinkawa, Ryo Miyake, Madoka Takai

## Main text

(iScience *26*, 107820; October 20, 2023)

In the originally published version of this article, the authors mistakenly identified a material used in the assembly of the microfluidic system. Specifically, all mentions of polydimethylsiloxane (PDMS) were corrected to polymethyl methacrylate (PMMA) in the main text and the legends of Figures 2 and S4. These corrections do not affect the experimental results, data interpretation, or conclusions of the article. The authors apologize for any confusion caused by these errors.

