# Supplementary material for: An automatic immuno-microfluidic system integrating electrospun polystyrene microfibrous reactors for rapid detection of salivary cortisol
Source: iScience. 2023 Sep 6;26(10):107820. doi: 10.1016/j.isci.2023.107820 (PMC10518708; doi:10.1016/j.isci.2023.107820)
Supplement: Document S1. Figures S1–S8 and Tables S1 and S2 [file mmc1.pdf]

iScience, Volume *26*

Supplemental information

An automatic immuno-microfluidic system integrating electrospun polystyrene microfibrous reactors for rapid detection of salivary cortisol

Yecan Wang, Hiroshi Murakami, Toshihiro Kasama, Shigenobu Mitsuzawa, Satoru Shinkawa, Ryo Miyake, and Madoka Takai

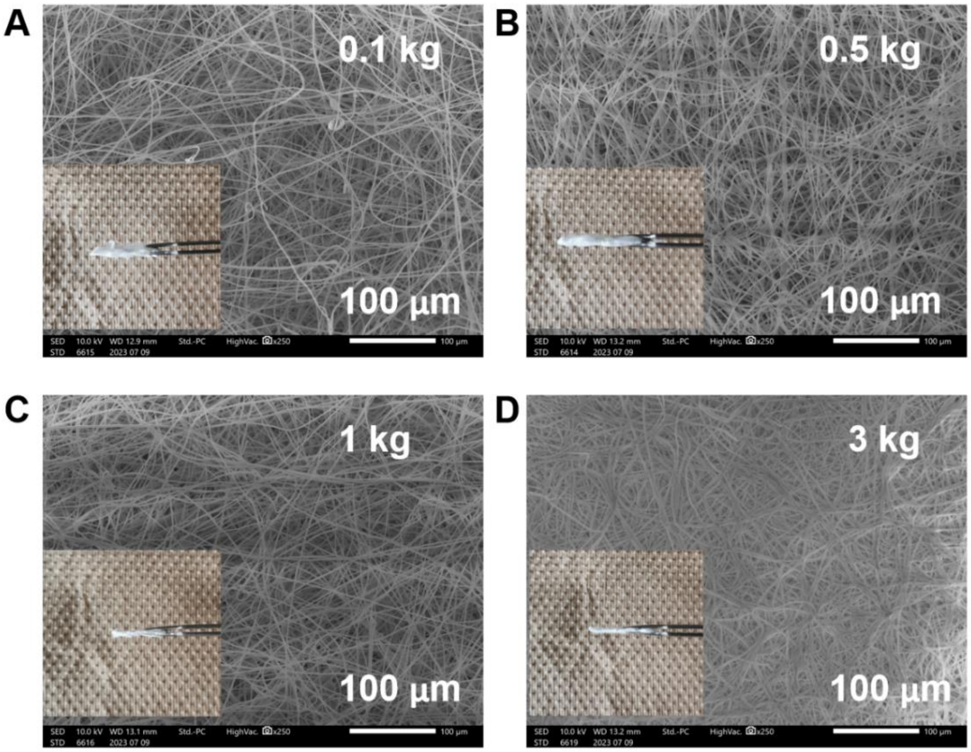

E

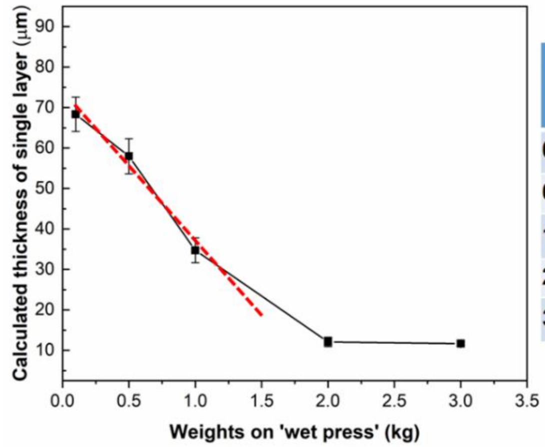

Weight (kg)	Thickness of 64-layered mats (mm)	Calculated thickness of single layer (µm)
**0.1**	4.37±0.27	68.33±4.22
**0.5**	3.71±0.28	57.97±4.37
**1**	2.22±0.20	34.74±3.08
**2**	0.77±0.07	12.08±1.14
**3**	0.75±0.03	11.67±0.41

Figure S1. Effects of pressure on the thickness of microfibers, Related to STAR Methods
(A)-(D) SEM images of microfiber mats wet pressured by 0.1 kg, 0.5 kg, 1 kg, and 3 kg weights, respectively. **(E)** The thicknesses of microfiber mat measured by 'wet press' technique with various compacting weights (0.1 kg, 0.5 kg, 1 kg, 2 kg, 3 kg) applied on. The red dashed line demonstrated the linearly fitted relationship between the applied pressure and the thickness of the microfibrous mat single layer. Error bars: standard error of the mean from n=3 independent measurements.

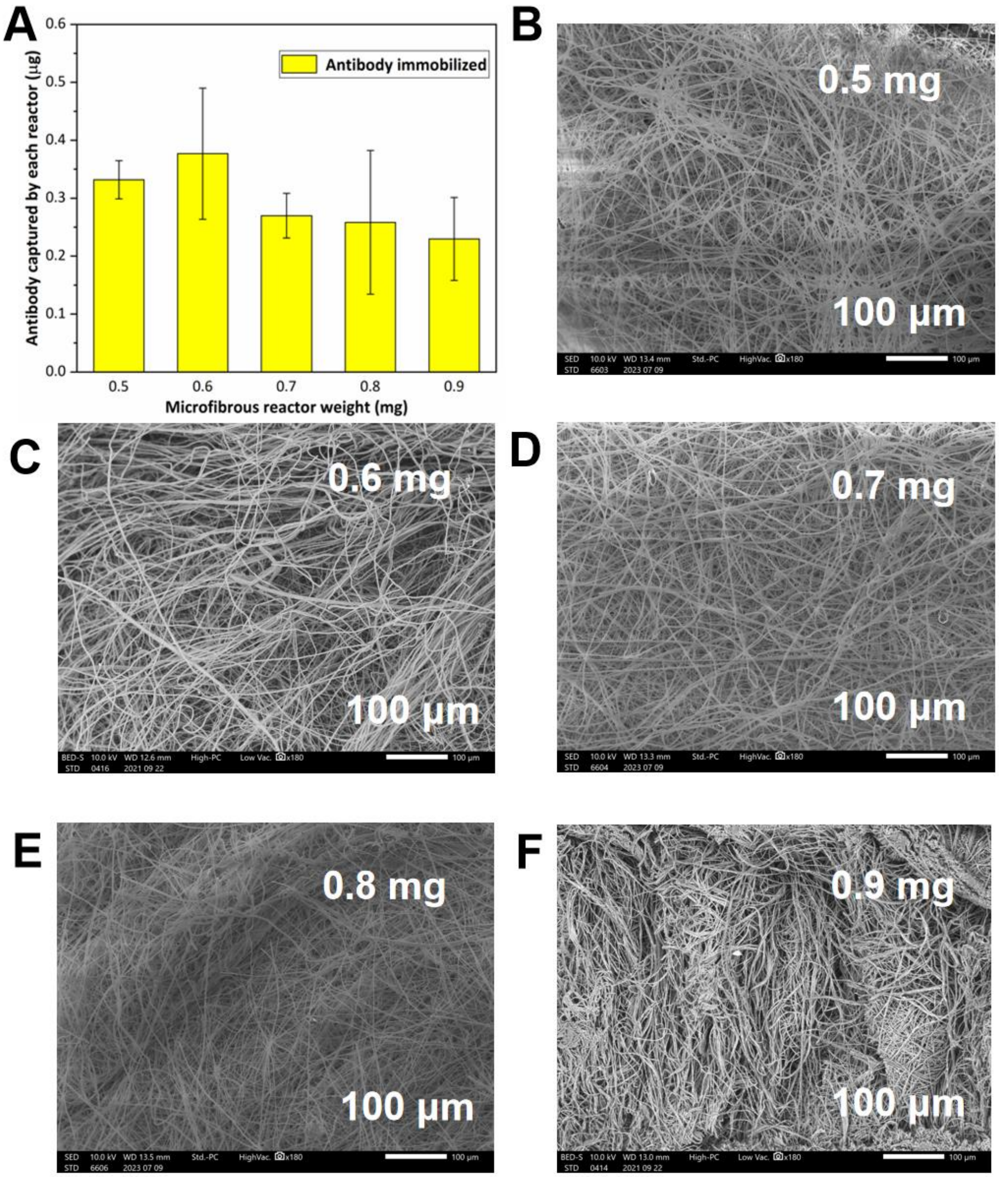

Figure S2. Capacity of antibody capture by microfibrous reactors with various weights, Related to STAR Method

(A) The evaluation of the antibody capture capacity of the microfibrous reactors. **(B)-(F)** The SEM images of the microfibrous reactors fabricated by rolled 8 cm×7 cm, 8 cm×8 cm, 8 cm×9 cm, 8 cm×10 cm, and 8 cm×11 cm microfibrous mats, respectively. Error bars: standard error of the mean from n=3 independent measurements.

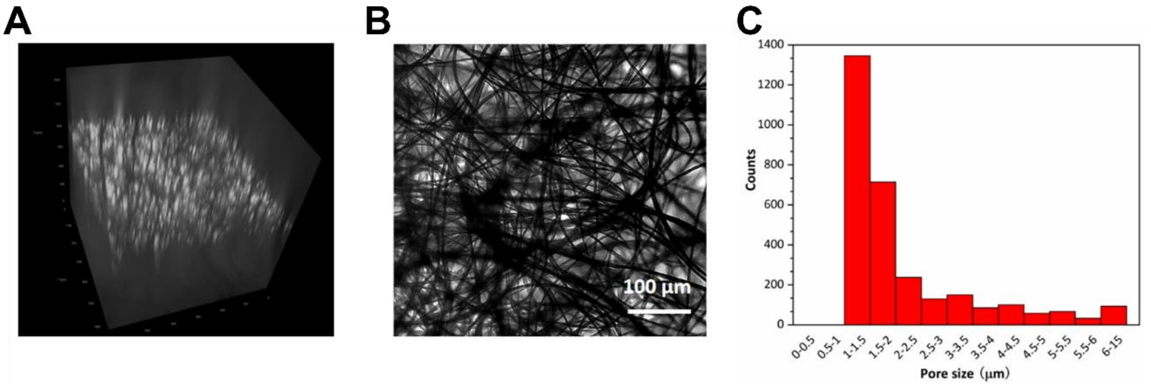

Figure S3. Characterization and analysis of the inner structure of the microfibrous reactor by confocal microscopy, Related to Figure 1 and STAR Method

(A) Confocal microscopic 3D reconstructed model of the microfibrous reactor inner structure.

(B) The home view of the projection image of one scanned slice with a thickness of 6.54 µm.

(C) The distribution of pore sizes in the microfibrous reactor.

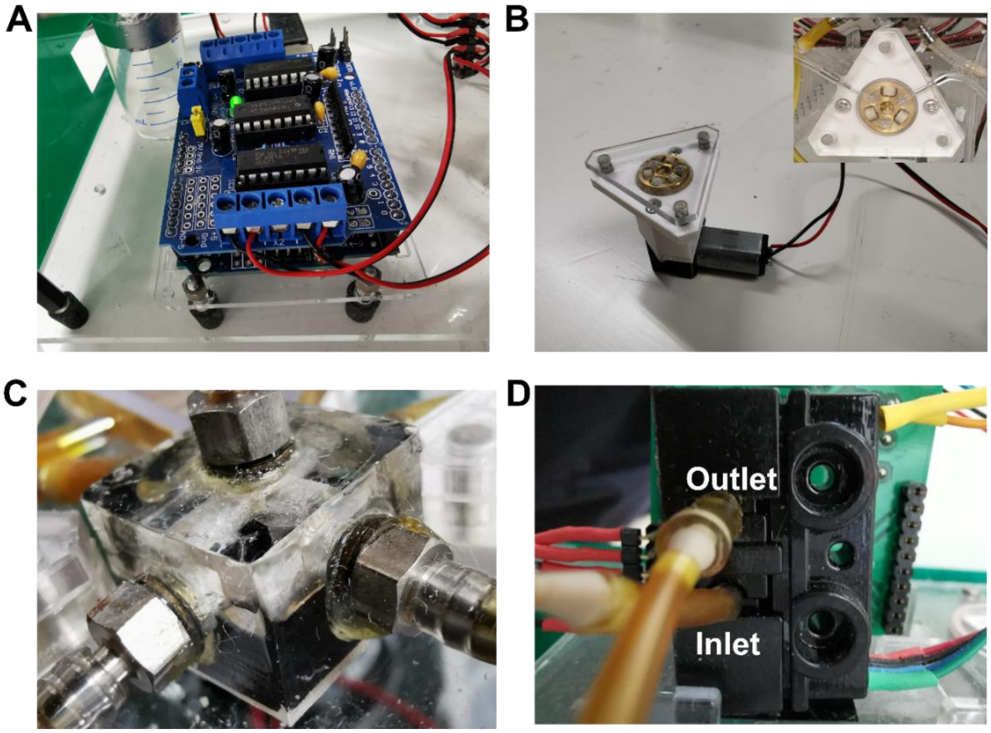

Figure S4. Components of the microfluidic system, Related to Figure 2

(A) Arduino circuit board for operation of software.

(B) Fluid delivery pumps.

(C) PDMS exchanger. We used a milling machine (PROXXON Corp., Osaka, Japan) to drill two cross-shaped channels (d=5mm) in a 15 mm ×15 mm× 10 mm PDMS bulk. Then the tubes were connected and fixed by metal joints.

(D) A 3D printer produced a 10 mm × 20 mm × 3.5 mm black resin cassette. The reagents were delivered into the cassette through the inlet (down) and out for the waster collector through the outlet (upper). The RGB photodetector was enclosed in the black cassette to prevent the effects of the external light.

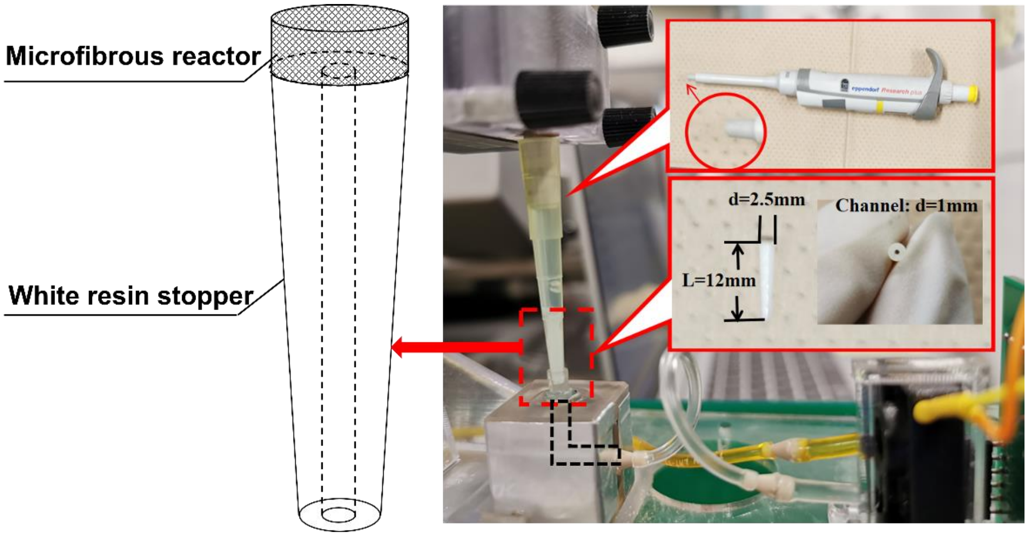

Figure S5. Structure of the commercial pipette tip with the microfibrous reactor, Related to Figure 2

200 μL pipette tip with a 3D printed white resin stopper. A tip cone was disassembled from a 200 μL pipettor (Eppendorf Corp., Germany) to be used as the joint to the Tygon tube. The stopper with a channel (d=1 mm) is designed to fix in the tip with a length of 12 mm by a 3D printer.

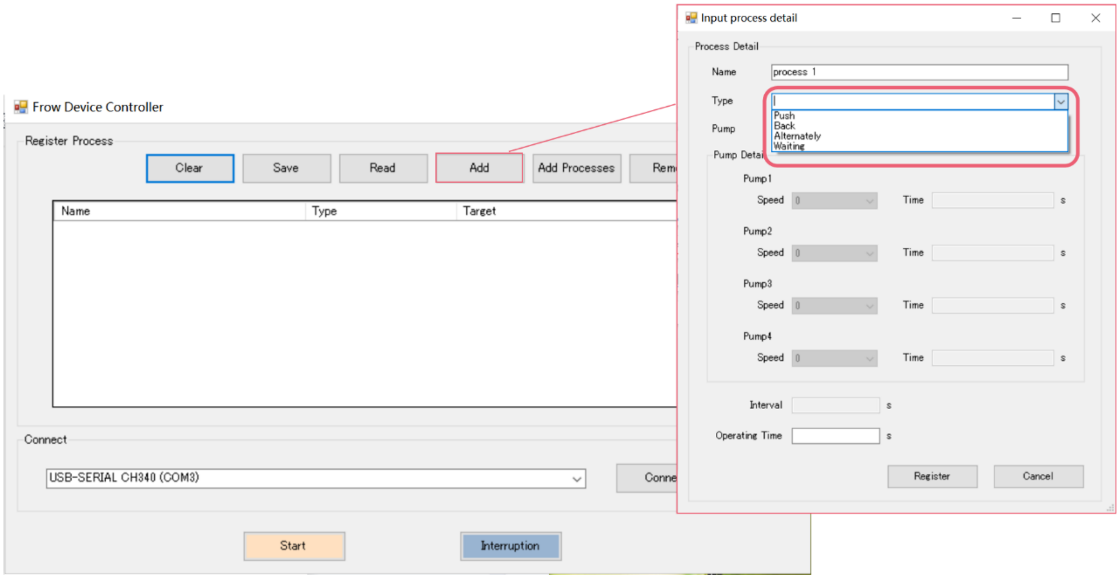

Figure S6. Window of the C++ developed software for the pumps control, Related to Figure 2 and the liquid feeding description

The process, including four modes (‘Push’, ‘Back’, ‘Alternate’, and ‘Waiting’) could be input and modified via this software.

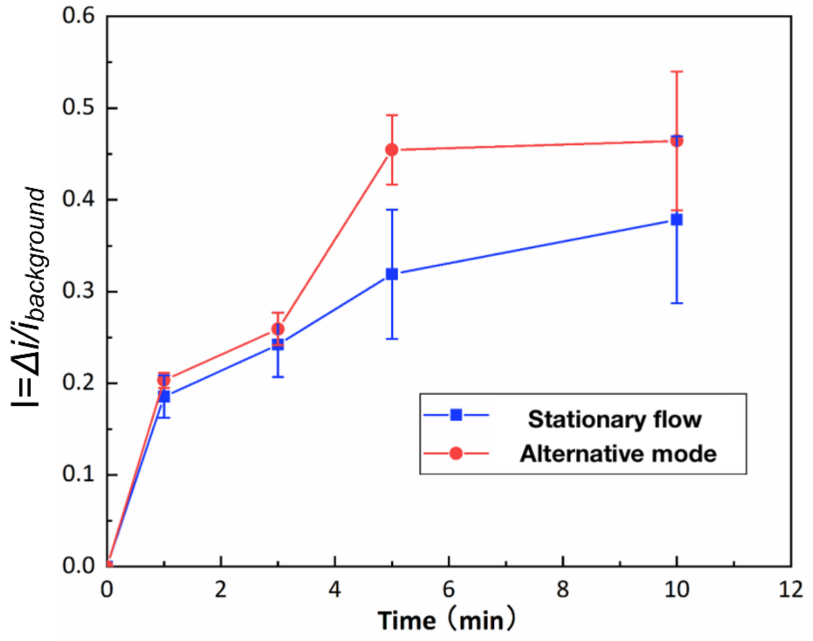

Figure S7. Relationship between signal output and TMB incubation time in stationary flow and 'Alternate mode', Related to Figure 2 and mass transport in the microfibrous reactor
The stationary flow represented that the fluid maintained stationary with no flow rate in the microfibrous reactor. The 'Alternate flow' applied reversal flow with a 255 μL/min flow rate. Error bars: standard error of the mean from n=3 independent measurements.

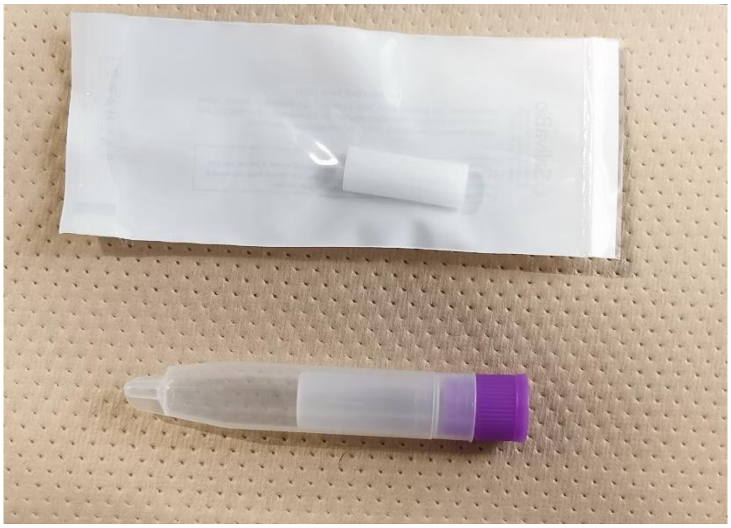

Figure S8. SalivaBio Oral Swab and a swab storage tube used for saliva harvest, Related to STAR Method

Table S1. B/B_0 and standard deviations obtained through the microfluidic system for standard curves plotting, Related to Figure 4

	HRP-cortisol : cortisol 150 μL:50 μL			HRP-cortisol : cortisol 200 μL:50 μL			HRP-cortisol : cortisol 200 μL:25 μL		
Concentration, ng/mL	B/B_0	Std	RSD, %	B/B_0	Std	RSD, %	B/B_0	Std	RSD, %
0.12	0.9869	0.1079	10.93	1.0131	0.0237	2.34	1.0592	0.0409	3.87
0.37	0.8748	0.0485	5.55	0.9693	0.0183	1.89	--	--	
1.1	0.6231	0.0483	5.55	0.8819	0.0519	5.88	0.9839	0.0615	6.25
3.3	0.5640	0.0236	7.76	0.7321	0.0455	6.21	0.8750	0.0630	7.20
10	0.4592	0.0523	4.18	0.5412	0.0319	5.89	0.6134	0.0129	2.10
30	0.3258	0.0638	11.38	0.3741	0.0441	11.80	0.3718	0.0462	12.43

Table S2. 5PL regression parameters of the fitted standard curves with determined HRP-cortisol: cortisol volumes (150 μL:50 μL, 200 μL:50 μL, and 200 μL:25 μL), Related to Figure 4

HRP-cortisol:cortisol		Conventional competitive ELISA kit		Microfluidic system	
μL : μL		5PL regression parameters	R^2	5PL regression parameters	R^2
150:50	*a*	0.97	0.9608	0.99	0.9582
	b	1.01		4.42	
	c	4.98		0.18	
	d	0.08		0.05	
	g	2.21		0.05	
200:50	*a*	0.97	0.9779	1.05	1
	b	0.78		0.81	
	c	84.91		6.05	
	d	0.07		0.16	
	g	11.57		0.91	
200:25	*a*	1.03	0.9686	1.06	0.9981
	b	1.02		1.07	
	c	974.27		18.44	
	d	0.08		0.23	
	g	148.96		1.84	
